# Nitrogen-Doped TiO_2_–C Composite Nanofibers with High-Capacity and Long-Cycle Life as Anode Materials for Sodium-Ion Batteries

**DOI:** 10.1007/s40820-018-0225-1

**Published:** 2018-10-09

**Authors:** Su Nie, Li Liu, Junfang Liu, Jianjun Xie, Yue Zhang, Jing Xia, Hanxiao Yan, Yiting Yuan, Xianyou Wang

**Affiliations:** 1 National Base for International Science and Technology Cooperation, National Local Joint Engineering Laboratory for Key Materials of New Energy Storage Battery, Hunan Province Key Laboratory of Electrochemical Energy Storage and Conversion, School of Chemistry, Xiangtan University, Xiangtan, 411105 People’s Republic of China; 2 Key Laboratory of Advanced Energy Materials Chemistry (Ministry of Education), Nankai University, Tianjin, 300071 People’s Republic of China

**Keywords:** Nanofibers, Anode materials, Sodium-ion batteries, Pseudocapacitance, Nitrogen-doping

## Abstract

Nitrogen-doped TiO_2_–C composite nanofibers (TiO_2_/N–C NFs) were manufactured by a convenient and green electrospinning technique in which urea acted as both the nitrogen source and a pore-forming agent. The TiO_2_/N–C NFs exhibit a large specific surface area (213.04 m^2^ g^−1^) and a suitable nitrogen content (5.37 wt%). The large specific surface area can increase the contribution of the extrinsic pseudocapacitance, which greatly enhances the rate capability. Further, the diffusion coefficient of sodium ions (*D*_Na+_) could be greatly improved by the incorporation of nitrogen atoms. Thus, the TiO_2_/N–C NFs display excellent electrochemical properties in Na-ion batteries. A TiO_2_/N–C NF anode delivers a high reversible discharge capacity of 265.8 mAh g^−1^ at 0.05 A g^−1^ and an outstanding long cycling performance even at a high current density (118.1 mAh g^−1^) with almost no capacity decay at 5 A g^−1^ over 2000 cycles. Therefore, this work sheds light on the application of TiO_2_-based materials in sodium-ion batteries.

**Electronic supplementary material:**

The online version of this article (10.1007/s40820-018-0225-1) contains supplementary material, which is available to authorized users.

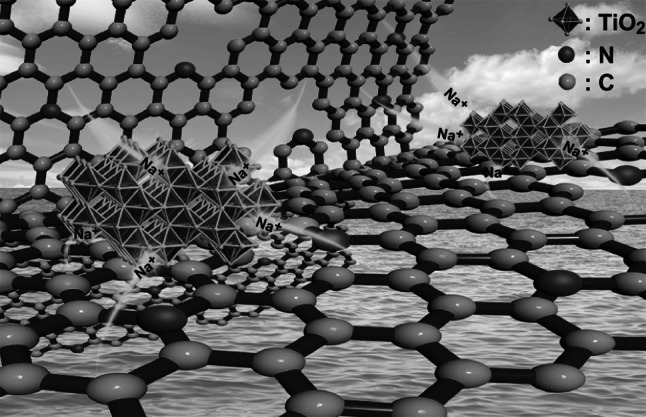

## Highlights

- Nitrogen-doped TiO_2_–C composite nanofibers (TiO_2_/N–C NFs) are fabricated using green, inexpensive urea as a nitrogen source and pore-forming agent.
- X-ray photoelectron spectroscopy results reveal changes in the content of different nitrogen species in detail.
- The TiO_2_/N–C NFs anode exhibits excellent sodium storage performance.

## Introduction

In recent decades, lithium-ion batteries (LIBs) play an important role in daily life (for example, in electric/hybrid vehicles and portable electronic products) owing to their excellent energy densities and long life spans [1–5]. Nevertheless, the disadvantages of limited lithium resources and high costs limit the commercial application of LIBs in large-scale energy storage. In contrast, sodium-ion batteries (SIBs) are more suitable for low-cost energy storage devices because of the abundance of sodium and affordable price [6–9]. Nevertheless, it is still challenging to find a suitable host material with a larger space suitable for sodium ion insertion/extraction, which is necessary because the Na^+^ ion (1.06 Å) is ca. 40% larger than the Li^+^ ion (0.76 Å) [10, 11]. Therefore, it is vital to investigate suitable electrode materials for SIBs.

There are many reports on anode materials for SIBs, including alloying/dealloying reaction materials (Sn, Sb) [12, 13], conversion reaction materials (FeS_2_, Fe_2_O_3_) [14, 15], and insertion/extraction reaction materials (Na_2_Ti_3_O_7_, TiO_2_) [16, 17]. In particular, anatase titanium dioxide (TiO_2_), with a high natural abundance, nontoxicity, a small volume change (less than 4%), and low production cost, has attracted extensive attention as a promising anode material for SIBs [18]. However, TiO_2_ has inherent defects, such as inferior electrical conductivity (10^−12^ S cm^−1^) as well as narrow ionic channels that cannot support rapid transfer of sodium ions [19–21], resulting in low specific capacity and serious capacity loss at high current densities. In order to improve its sodium storage performance, an important strategy is to increase its conductivity. One typical approach is to decrease the size of TiO_2_ particles or design novel nanostructures such as nanowires [22], nanospheres [23], or nanotubes [24], which can greatly shorten the sodium ion diffusion distance and promote electronic transport. Another effective method is recombination with carbon or doping of multivalent ions with Fe [25], S [26], Nb [27], or N [28].

Recently, nitrogen doping has been reported as an effective method to increase both the electronic and ionic conductivities of bulk materials [29, 30]. Nitrogen-doped carbon hollow spheres and carbon nanofibers (NFs) have exhibited excellent electrochemical properties as anode materials for SIBs [31, 32]. Nitrogen doping is effective not only for carbon materials, but also for transition-metal-oxide-based carbon composites. Some nitrogen-doped carbon composite transition metal oxides (such as MnO [33], Fe_2_O_3_ [34], Co_3_O_4_ [35], and TiO_2_ [36]) have been reported and showed satisfactory results. However, at present, the common methods of introducing N atoms are to calcine bulk materials in a poisonous atmosphere of N_2_/NH_3_ or to use rare and expensive nitrogen-rich materials, such as 3-hydroxytyramine hydrochloride, diethylenetriamine, polyaniline, and polypyrrole as nitrogen sources.

As a convenient and universal technology for producing polymers or composite material NFs, the electrospinning method has been widely applied in both academic research and industrial applications. Very recently, there have been many studies on the preparation of high-performance electrode materials (such as SnS/C, Na_2_VPO_4_F/C, and NiO/C) by electrospinning technology [37–39]. The obtained one-dimensional NFs with high specific surface areas can provide facile electronic and ionic transport. Further, the porous structure is highly tolerant of stress changes during the reaction in the battery, making it conducive to the realization of a long cycle life [39, 40].

Herein, a simple, economical, and green electrospinning process is proposed to obtain nitrogen-doped TiO_2_–C composite NFs (denoted as TiO_2_/N–C NFs). Inexpensive urea is used as the nitrogen source and pore-forming agent. Owing to the advantages of nitrogen doping and the large specific surface area, a TiO_2_/N–C NF electrode displays outstanding electrochemical properties.

## Experimental Section

### Synthesis of Materials

The TiO_2_/N–C NFs were synthesized by electrospinning followed by high-temperature carbonization. The precursor solution for electrospinning was made as follows: First, 5.0 mL of *N*, *N*-dimethylformamide (Kermel, 99.5%) and 1.05 g of glacial acetic acid (CH_3_COOH, Kermel, 99.5%) were mixed; then, 0.1 g of urea [CO(NH_2_)_2_, Kermel, 99.5%] and 0.97 g of tetra-n-butyl titanate (C_16_H_36_O_4_Ti, Kermel, 99%) were added with stirring. Next, 0.4 g of polyvinylpyrrolidone (PVP, Mw = 1,300,000, Alfa Aesar) was added to the above mixed solution under stirring for 12 h to acquire a clear precursor solution. The obtained solution was injected into a 10-mL syringe connected to a blunt-tip needle and spun on an electrospinning unit with an applied voltage of 14 kV. The distance between the needle and the collector was set to 14 cm, and the flow velocity was 0.36 mL h^−1^. The collected NFs were dried at 70 °C for 8 h in a vacuum oven and then precalcined at 200 °C for 2 h. Finally, the TiO_2_/N–C composite NFs were obtained by calcination at 550 °C for 4 h in an inert atmosphere of Ar, where the ramping rate was set to 4 °C min^−1^.

For comparison, the pristine TiO_2_–C NFs and the other two types of TiO_2_/N–C NFs with different N contents were prepared using similar methods by adjusting the amount of urea to 0, 0.05, and 0.2 g, respectively.

### Structural Characterization

The as-prepared materials were examined by X-ray diffraction (XRD) in a Rigaku D/Max-2500 powder diffractometer with Cu *K*α radiation (*λ* = 1.5418 Å). The morphologies of the synthesized samples were observed using scanning electron microscopy (SEM, JEOL, SM-71480) and transmission electron microscopy (TEM, JEOL, JEM-100CX). The chemical composition of the as-prepared materials was analyzed using X-ray photoelectron spectroscopy (XPS, ThermoFisher, K-Alpha^+^). N_2_ adsorption–desorption isotherms were obtained using TriStar II 3020 (Micromeritics, USA) at liquid nitrogen temperature (77.3 K). The specific surface area (*S*_BET_) was calculated by the conventional Brunauer–Emmett–Teller (BET) method. Thermogravimetry was performed using a TGA Q50 (TA Instruments) analyzer. Raman spectra were obtained using a Raman spectrometer (Renishaw, Model 1000) at an excitation wavelength of 514 nm.

### Electrochemical Measurements

Polyvinylidene fluoride binder (10 wt%), 20 wt% carbon black, and 70 wt% active material (TiO_2_/N–C NFs or TiO_2_–C NFs) were dissolved in an appropriate amount of *N*-methyl-2-pyrrolidinone. The obtained slurry was evenly coated on copper foil and placed in a vacuum oven at 110 °C for 12 h. Circular pieces 1 cm in diameter were punched from the dried copper foil and used as working electrodes; their mass load was 1.2 ± 0.2 mg cm^−2^. In an argon-filled glove box, CR2025-type coin cells were assembled; metallic sodium was used as the counter electrode and separated from the work electrode by a glass fiber (Whatman, GF/C). The electrolyte was a solution of 1 mol L^−1^ NaClO_4_ dissolved in propylene carbonate/ethylene carbonate (1:1 by volume). The coin cells were cycled in galvanostatic discharge–charge measurements using a battery testing system (Neware, China) at room temperature at voltage intervals of 0.01 and 2.5 V. Both cyclic voltammetry (CV) tests and electrochemical impedance spectroscopy (EIS) experiments were conducted on a CHI660E electrochemistry workstation (Chenhua, Shanghai).

## Results and Discussion

**Fig. 1:**
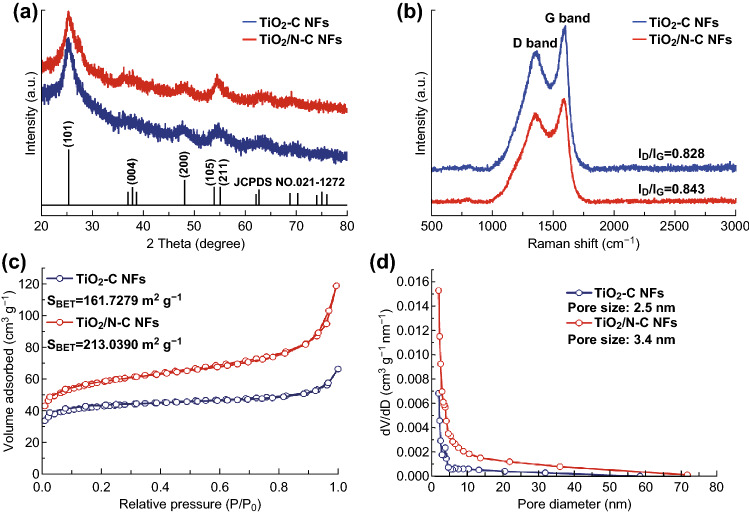
**a** XRD patterns and **b** Raman spectra of TiO_2_/N–C NFs and TiO_2_–C NFs. **c** N_2_ adsorption–desorption isotherms and **d** the corresponding pore size distributions of TiO_2_/N–C NFs and TiO_2_–C NFs

Figure 1a shows the XRD patterns of the TiO_2_/N–C NFs and TiO_2_–C NFs. All the peaks of both the TiO_2_/N–C NFs and TiO_2_–C NFs are in good agreement with those of anatase TiO_2_ (JCPDS No. 21-1272). No peaks from other phases were observed, demonstrating the high purity of the as-prepared samples. The weak intensity of the TiO_2_ peaks may be attributed to TiO_2_ nanoparticles embedded in an amorphous carbon matrix, which will be further demonstrated in the TEM results below [18]. The Raman spectra in Fig. 1b reveal two obvious peaks at 1350 and 1600 cm^−1^, which correspond to a disorder-induced feature (the D band) and the *E*_2g_ mode of graphite (the G band), respectively. An intensity ratio of the D/G bands (*I*_D_/*I*_G_ < 1.0) can be used to identify a certain degree of graphitization in a carbon matrix [41–44]. The *I*_D_/*I*_G_ values of the TiO_2_/N–C NFs and TiO_2_–C NFs are 0.843 and 0.828, respectively, which indicate high-quality electrical conductivity. In addition, a slight increase in the value of *I*_D_/*I*_G_ (which corresponds to increasing disorder) may be related to the introduction of nitrogen, which caused defects and disordered structure in the carbon layer.

N_2_ adsorption–desorption measurements were taken to determine the BET surface area and pore distribution of the TiO_2_/N–C NFs and TiO_2_–C NFs. In Fig. 1c, d, the BET surface area and average pore width of the TiO_2_/N–C NFs are 213.04 m^2^ g^−1^ and 3.4 nm, whereas the TiO_2_–C NFs show a BET surface area and average pore width of 161.3 m^2^ g^−1^ and 2.5 nm, respectively. The increased specific surface area and average pore width may be due to decomposition of urea during the heating process, as shown in Eq. 1:

1 $${\text{CO}}\left( {{\text{NH}}_{ 2} } \right)_{ 2} \mathop{\longrightarrow}\limits^{\Delta } {\text{NH}}_{ 3} \uparrow + {\text{HCNO}}$$

The larger specific surface area ensures full infiltration of the active material and electrolyte, thereby shortening the transport path to accelerate the rapid transfer of Na^+^/e^−^ [45]. The TiO_2_ content of the composites was determined by TGA. As shown in Fig. S1, the weight losses of the composites are ~ 28.9% and 26.4% in air, which implies a TiO_2_ content of 71.1 wt% in the TiO_2_/N–C NFs and 73.6 wt% in the TiO_2_–C NFs.

**Fig. 2:**
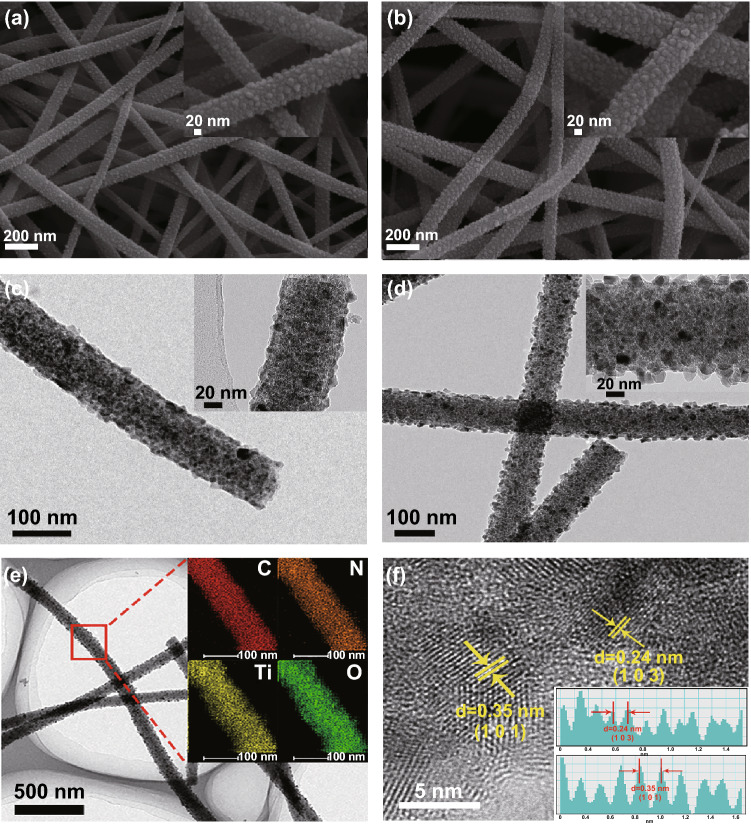
SEM images of **a** TiO_2_–C NFs and **b** TiO_2_/N–C NFs. TEM images of **c** TiO_2_–C NFs and **d** TiO_2_/N–C NFs. **e** TEM elemental mapping images of C, N, Ti, and O in TiO_2_/N–C NFs. **f** HRTEM image of TiO_2_/N–C NFs with lattice spacing (the inset shows statistical tables of the interplanar crystal spacing)

Figure 2a, b shows SEM images of the TiO_2_–C NFs and TiO_2_/N–C NFs, respectively. There is no obvious difference in the structural features of the TiO_2_–C NFs and TiO_2_/N–C NFs. Both samples show rough NFs with diameters from 100 to 130 nm. The structures of the TiO_2_–C NFs and TiO_2_/N–C NFs were further analyzed by TEM (Fig. 2c, d). The results show that TiO_2_ particles are appropriately buried in the carbon matrix to develop a stable NF structure. Further, it can be seen, as intuitively expected, that the surface of the TiO_2_/N–C NFs is rougher than that of the TiO_2_–C NFs, so it can provide a larger specific surface area to help release more of the pseudocapacitor from the electrode materials [16]. The TEM mapping images of the TiO_2_/N–C NFs in Fig. 2e reveal that C, N, Ti, and O are uniformly dispersed in the NFs. The high-resolution TEM (HRTEM) images in Fig. 2f clearly show lattice fringes of 0.35 and 0.24 nm in the nanoparticles, which correspond to the (101) and (103) planes of anatase-phase TiO_2_, respectively.

**Fig. 3:**
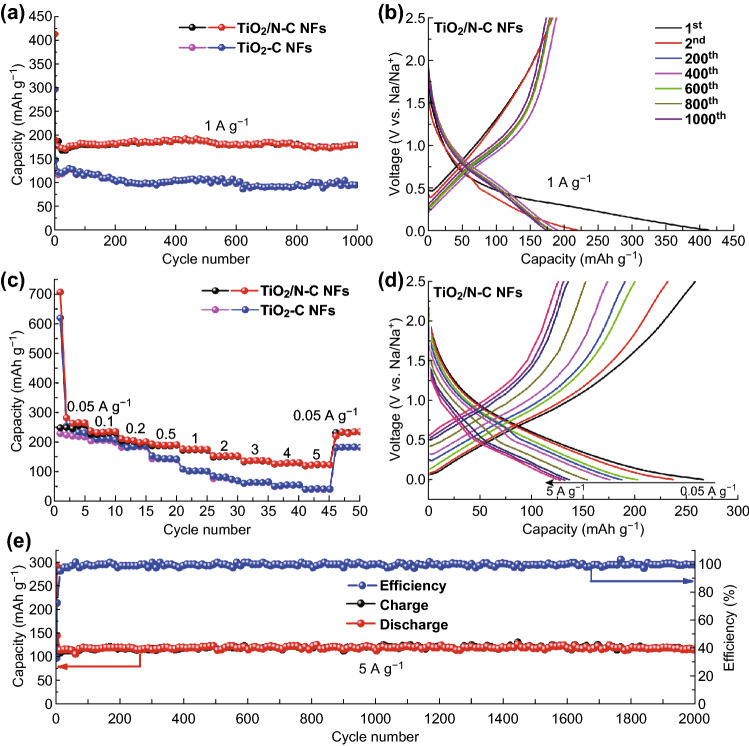
**a** Cycling performance of the TiO_2_/N–C NF and TiO_2_–C NF electrodes under a current density of 1 A g^−1^. **b** Continuous discharge and charge curves of TiO_2_/N–C NF electrode under a current density of 1 A g^−1^. **c** Rate capability of TiO_2_/N–C NFs and TiO_2_/C NFs. **d** Charge–discharge curves of TiO_2_/N–C NFs at 0.05–5 A g^−1^ in the range of 0.01–2.5 V. **e** Long-term cycle performance of TiO_2_/N–C NFs under a high current density of 5 A g^−1^

The electrochemical performance was assessed by galvanostatic discharge–charge measurement in sodium half-cells. Figure 3a reveals the cycling properties of the TiO_2_/N–C NF and TiO_2_–C NF electrodes at 1 A g^−1^. It is observed that the reversible discharge capacity of the TiO_2_–C NF electrode can be maintained only at 94.9 mAh g^−1^ after 1000 cycles, and the corresponding capacity retention rate is 64.3% (relative to the discharge capacity of the second cycle). Under the same condition, the TiO_2_/N–C NF electrode displays a much higher discharge capacity of 179.2 mAh g^−1^, with a satisfactory capacity retention of 94.7%. The corresponding voltage curves of TiO_2_/N–C NFs in different cycles are exhibited in Fig. 3b. The large capacity loss in the first cycle is associated mainly with the interface reaction between the active materials and electrolyte, which leads to the generation of a solid electrolyte interface (SEI) film [45, 46]. Further, as the number of cycles increases, the charge and discharge platform of the TiO_2_/N–C NFs shows no significant change with respect to that of the TiO_2_–C NFs (Fig. S2a), indicating excellent cycling stability.

Figure 3c shows the rate performance of the two samples. The TiO_2_/N–C NF electrode could release reversible capacities of 265.8, 236.8, 202.4, 187.2, 175.6, 153.7, 136.4, and 132.1 mAh g^−1^ at current densities of 0.05, 0.1, 0.2, 0.5, 1, 2, 3, and 4 A g^−1^, respectively. Even at 5 A g^−1^, a reversible capacity of 124.5 mAh g^−1^ could be achieved. The discharge capacity could be maintained at 236.2 mAh g^−1^ when the current density recovered to 0.05 A g^−1^, which represents an excellent rate capability. In contrast to that of the TiO_2_/N–C NFs, the capacity of the TiO_2_–C NFs decreased significantly as the current density increased and dropped to 43.1 mAh g^−1^ at 5 A g^−1^. The outstanding rate properties may be due mainly to the improved conductivity resulting from the incorporation of N atoms. Figure 3d shows the corresponding discharge–charge curves. The discharge capacity of the TiO_2_/N–C NFs gradually decreases with an increase in current density. Nonetheless, the TiO_2_/N–C NF anode reveals less polarization than the TiO_2_–C NF anode (Fig. S2b), which further demonstrates the excellent rate capability. An ultra-long-term high-rate cycling performance test was performed to further verify the electrochemical performance of the TiO_2_/N–C NF anode. As shown in Fig. 3e, the specific capacity of the TiO_2_/N–C NF anode remains at 118.1 mAh g^−1^ after 2000 cycles at 5 A g^−1^ and exhibits almost no capacity decay. In order to explore the effect of adding urea to the precursor solution on the TiO_2_/N–C NF anode, the electrochemical properties of samples with different amounts of urea (0.05, 0.1, and 0.2 g) are shown in Fig. S3. The composite with 0.1 g of added urea obviously exhibits the best cycle stability and rate performance.

In order to determine the mechanism of the outstanding cycling stability of the TiO_2_/N–C NF electrode, a Na half-cell tested at a current density of 1 A g^−1^ for 1000 cycles was disassembled, and the morphology and microstructure of the TiO_2_/N–C NF electrode after cycling were observed by TEM, as shown in Fig. S4a, b. The morphology of the TiO_2_/N–C NFs remained essentially integrated. Further, as shown in the energy-dispersive X-ray spectroscopy (EDS) elemental mapping images in Fig. S4b, C, N, Ti, and O were still uniformly distributed in the NFs after a long cycling duration, indicating the mechanical stability of the fibers. Furthermore, the presence of Na in the EDS element mapping images also illustrates the process of sodium insertion/extraction during the cycle. An HRTEM image of the TiO_2_/N–C NFs after 1000 cycles further reveals that the crystal structure of the TiO_2_/N–C NFs remained integrated. From the above discussion, the preservation of the morphology and crystal structure of the TiO_2_/N–C NF electrode after cycling further explains the excellent electrochemical properties.

**Fig. 4:**
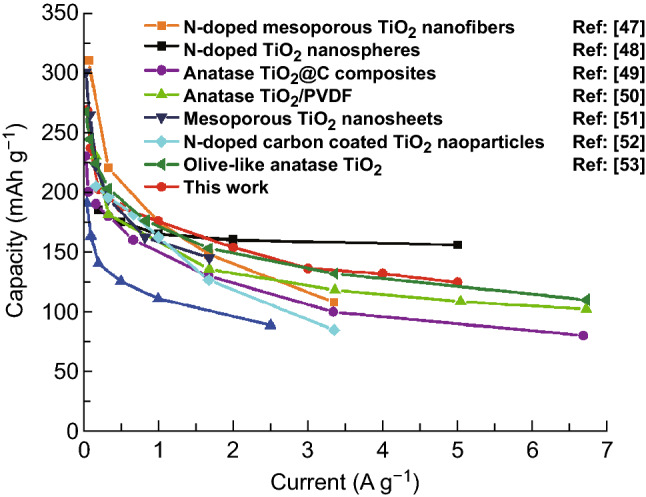
Comparison of rate capability of TiO_2_/N–C NFs with that of previously reported TiO_2_-based composites with various morphologies

Moreover, the rate capability of the TiO_2_/N–C NFs is comparable with the previously reported results for many other TiO_2_-based materials [47–53], which are presented in Fig. 4. It is clear that the TiO_2_/N–C NFs in this work reveal a higher discharge capacity than most of the previously reported TiO_2_-based materials at the same current density. Although a nitrogen-doped TiO_2_ nanosphere anode shows higher discharge capacity at large current density, its cycle performance is inferior to that reported in this work [48]. The details are summarized in Table S1.

**Fig. 5:**
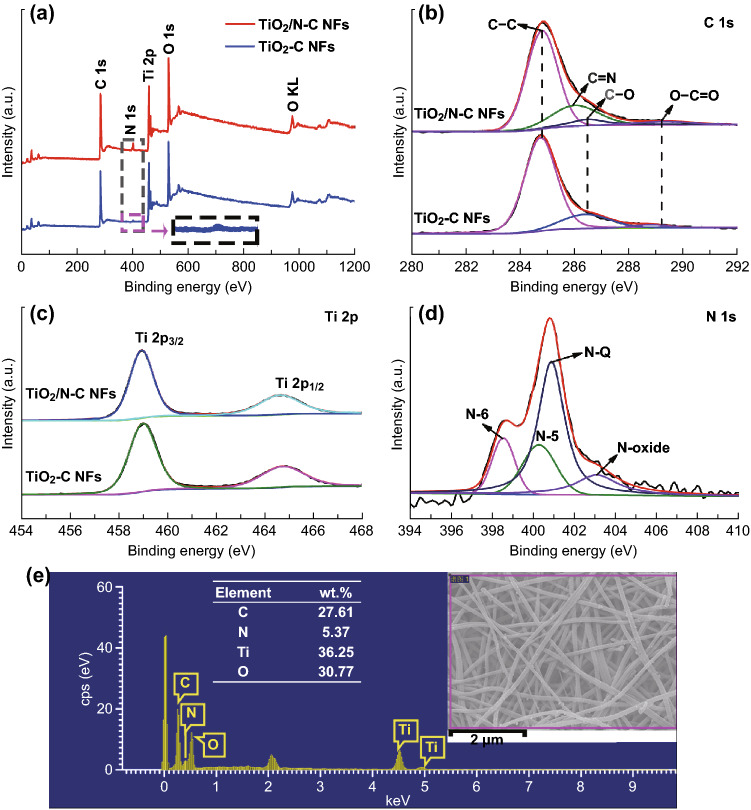
**a** XPS wide scan spectra of TiO_2_–C NFs and TiO_2_/N–C NFs. High-resolution **b** C 1*s* and **c** Ti 2*p* XPS spectra of TiO_2_–C NFs and TiO_2_/N–C NFs. **d** High-resolution N 1*s* spectra of TiO_2_/N–C NFs. **e** EDS spectrum of TiO_2_/N–C NFs

**Fig. 6:**
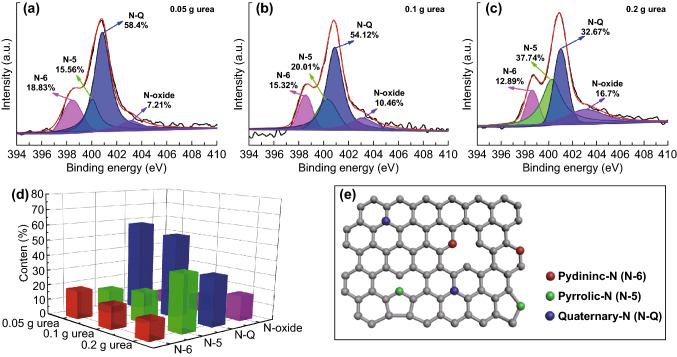
N 1*s* fine spectra of TiO_2_/N–C NFs with **a** 0.05 g, **b** 0.1 g, and **c** 0.2 g of added urea. **d** Histogram of ratio of different N species in various TiO_2_/N–C NF samples. **e** Schematic of structural binding conditions of nitrogen

XPS was used to investigate the surface composition and chemical states of the samples. The survey XPS spectrum (Fig. 5a) shows the presence of C, N, Ti, and O in the two samples, and the N peak of the TiO_2_/N–C NFs is significantly stronger than that of the TiO_2_–C NFs, confirming the successful introduction of N atoms by nitrogen doping. To further understand the detailed information on each element, high-resolution C 1*s*, N 1*s*, and Ti 2*p* spectra of the TiO_2_/N–C NFs are shown in Fig. 5b–d. In the C 1*s* region (Fig. 5b), both the TiO_2_/N–C NF and TiO_2_–C NF samples have three peaks at 284.84, 286.57, and 289.13 eV, which could correspond to C–C, C–O, and O–C=O, respectively [54, 55]. The main source of the C–O and O–C=O peaks is incomplete carbonization of PVP [56, 57]. Note that there is an additional peak at 286.04 eV corresponding to C=N for the TiO_2_/N–C NFs, which further confirms the successful introduction of nitrogen [11]. In addition, the state of nitrogen from carbonization of PVP is significantly different from that in the TiO_2_/N–C NFs (Fig. S5). It is obvious that the N-oxide peak does not appear in the spectrum of the TiO_2_–C NFs, possibly because the N content of the composite is too small. Thus, the introduction of urea does change the content and state of nitrogen significantly. As shown in Fig. 5c, peaks situated at approximately 458.97 and 464.67 eV could be observed for the two samples, indicating the presence of Ti^4+^ in TiO_2_ [21, 58]. In the N 1*s* spectrum of the TiO_2_/N–C NFs (Fig. 5d), four forms of nitrogen in carbon can be observed: pyridinic N (N-6) at 398.47 eV, pyrrolic/pyridone N (N-5) at 400.05 eV, quaternary N (N-Q) at 401.01 eV, and pyridine-N-oxide at 403.19 eV [59, 60]. These different types of nitrogen are shown schematically in Fig. 6e. Quaternary N located inside the graphene layer is also called graphitic N and will improve the electric conductivity of the carbon layer because it can provide excess free electrons. Other N atoms including pyrrolic N, pyridinic N, and oxidized N atoms, located at the edge or in the defects of the carbon layer, can provide active sites for Na^+^ insertion to enhance the Na^+^ storage capacity [61, 62]. The elemental distribution in the TiO_2_/N–C NFs was further investigated using the EDS spectrum (Fig. 5e), and the nitrogen content of the TiO_2_/N–C NFs was found to be 5.37 wt%.

Figure 6a–c shows the nitrogen content of TiO_2_/N–C NFs with different amounts of added urea. As shown in Fig. 6d, it is clear that the N-6 and N-Q contents continue to decline and the N-5 and N-oxide contents constantly increase with an increase in N content in the carbon layer. Along with the total N content, the specific percentages of diverse N species in various TiO_2_/N–C NF materials are displayed in Table 1. Specifically, TiO_2_/N–C NFs with 0.1 g of added urea have the highest N-Q content, which can ensure excellent conductivity in the electrode material. This may be the main reason that this sample shows the best electrochemical performance among the samples with different amounts of added urea, as shown in Fig. S3.

**Table 1:** Nitrogen content of samples to which different amounts of urea were added

Nitrogen content (wt%)					
Sample	Total (%)	N-6	N-5	N-Q	N-oxide
N–TiO_2_/C NFs (0.05 g urea)	4.64	0.87	0.72	2.71	0.34
N–TiO_2_/C NFs (0.1 g urea)	5.37	0.82	1.07	2.92	0.56
N–TiO_2_/C NFs (0.2 g urea)	6.02	0.77	2.27	1.97	1.01

**Fig. 7:**
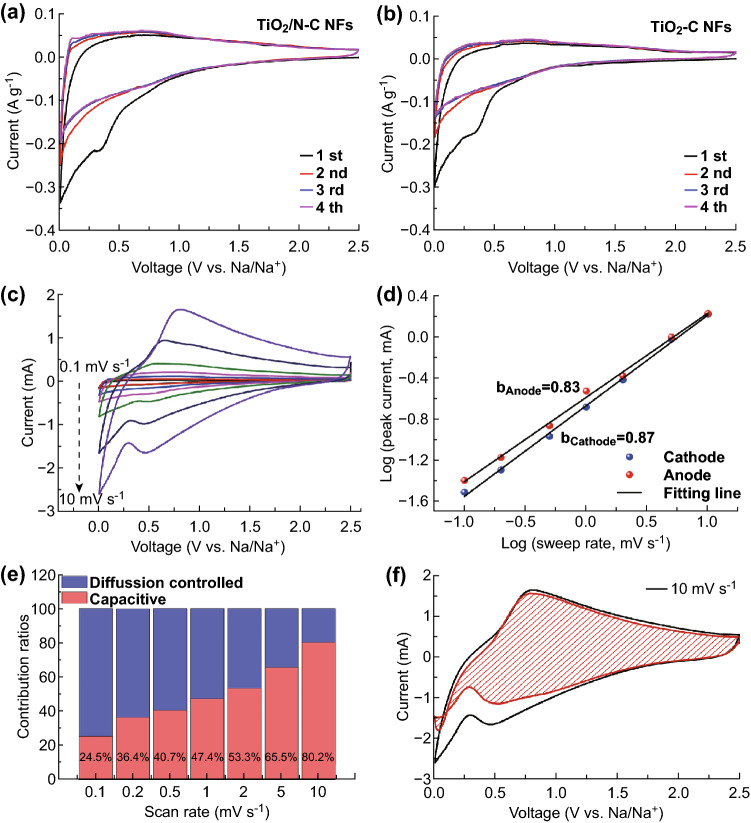
CV curves of **a** the TiO_2_/N–C NF electrode and **b** the TiO_2_–C NF electrode at a scan rate of 0.1 mV s^−1^. **c** CV plots of TiO_2_/N–C NFs at various sweep rates from 0.1 to 10 mV s^−1^. **d** Relationship between the peak currents and scan rates in logarithmic scale. **e** Diagram of capacitive contribution to the total capacity of TiO_2_/N–C NFs at different scan rates. **f** CV curve of TiO_2_/N–C NFs (black curve) and capacitive contribution measured at 10 mV s^−1^ (red-shaded region). (Color figure online)

The CV curves of the TiO_2_/N–C NF and TiO_2_–C NF electrodes at 0.1 mV s^−1^ are shown in Fig. 7a, b, respectively. Both of the electrodes show a pair of wide anodic/cathodic peaks at a potential of 0.76/0.63 V, which corresponds to Na^+^ extraction/insertion to/from the anatase TiO_2_ [48]. Further, there is a large gap between the first cycle and the next few cycles, which can be attributed to the generation of an SEI by decomposition of the electrolyte in the initial cycle, resulting in a low coulombic efficiency in the initial charge–discharge process [45]. The shape and intensity of the curves are highly consistent after the second cycle, implying satisfactory stability of these materials.

CV curves at scan rates of 0.1–10 mV s^−1^ were obtained to investigate the kinetic behaviors of the TiO_2_/N–C NFs (Fig. 7c). Obvious distortion from the basic shape can be found in the CV curves as the sweep rate increases. Several factors, such as ohmic resistance and/or diffusion constraints, are responsible for the distortion. Figure 7d shows the linear relationship between the logarithms of the sweep rates and the redox peak currents related to Na^+^ insertion/extraction in the TiO_2_/N–C NFs. The dominant charge storage mechanism is given by Eq. 2:

2 $$i = av^{b}$$

In this equation, the response current (*i*) and scan rate (*v*) are subject to a power-law relationship, and the value of *b* can be obtained from the slope (lg *i* vs. lg *v*). A *b* value of 0.5 indicates a diffusion-dominated process, and a *b* value of 1 suggests a capacitive-controlled process [63, 64]. The *b* values of both the cathodic (0.87) and anodic (0.83) peaks are larger than 0.7, indicating a pseudocapacitive process between the typical behaviors of batteries and capacitors [65]. Further, the contribution of these two mechanisms to the total charge storage can be determined by Eqs. 3 and 4:

3 $$i = k_{1} v + k{}_{2}v^{1/2}$$

4 $${\text{or}}\;i/v^{1/2} = k_{1} v^{1/2} + k_{2}$$

where *k*_1_*v* represents the contribution of the surface capacitive effects, and *k*_2_*v*^1/2^ corresponds to the contribution of intercalation/deintercalation effects. The fraction of the current response from these contributions to the specific potentials can be quantified by determining *k*_1_ and *k*_2_.

As the pseudocapacitive energy storage occurs on the surface or near the surface of the electrode, the ion diffusion is a type of diffusion on the surface or in the liquid phase, which has a much faster velocity than that in the solid phase. The characteristics of rapid ion diffusion greatly increase the ability of electrode materials to charge and discharge rapidly at high current densities [63, 66–68]. As shown in Fig. 7e, the contribution of the pseudocapacitance to the overall capacity increases with an increase in scan rate. Specifically, the contribution of the capacitive effect to the total charge stored at 0.1 mV s^−1^ is 24.5% and increases to 80.2% at 10 mV s^−1^ (Fig. 7f). However, the TiO_2_–C NFs show a low pseudocapacitance contribution of 16.2% at 0.1 mV s^−1^, and the value is only 67.4% even at 10 mV s^−1^ (Fig. S3). The large contribution of the pseudocapacitive contribution to the overall capacity may be correlated with the large specific surface area and the participation of nitrogen, which cause the TiO_2_/N–C NFs to exhibit excellent electrochemical performance as an anode in SIBs at ultra-high current density.

**Fig. 8:**
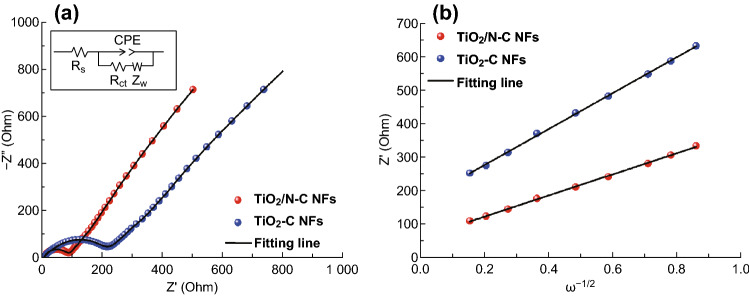
**a** Nyquist plots of TiO_2_/N–C NF and TiO_2_–C NF electrodes measured after the first cycle at 0.05 A g^−1^. (The inset shows the equivalent circuit model.) **b** Relationship between *Z*′ and *ω*^−1/2^ at low frequency

The dynamical properties of the TiO_2_/N–C NF electrode were explored through EIS measurement. As shown in Fig. 8a, the EIS patterns consist mainly of three parts: a small intercept in the high-frequency region (*R*_s_), a semicircle in the high-frequency region (*R*_ct_), and a sloping line in the low-frequency region (*Z*_w_). *R*_s_, *R*_ct_, and *Z*_w_ represent the resistance of the electrolyte in contact with particles, the charge transfer resistance, and Na^+^ ion diffusion in the anode active material, respectively [69, 70]. According to the fitted experimental data, the TiO_2_/N–C NFs show a lower *R*_ct_ value (85.5 Ω) than the TiO_2_–C NFs (210.3 Ω), indicating that the introduction of nitrogen can enhance the kinetics of the electrochemical reaction according to Eq. 5

5 $$i_{0} = RT /nFR_{\text{ct}}$$

where *i*_0_ represents the exchange current density, *R* is the gas constant, *T* is the temperature in Kelvin, *F* is the Faraday constant, and *n* is the number of electrons per molecule during the electronic transfer reaction. The value of *i*_0_ can be used to measure the resistance of the electrode (the higher the value, the lower the electrical resistance) [71]. The diffusion coefficient of sodium ions (*D*_Na+_) can be calculated using Eqs. 6 and 7:

6 $$D_{{{\text{Na}}^{ + } }} = \frac{{R^{2} T^{2} }}{{2A^{2} n^{4} F^{4} c^{2} \sigma_{\text{w}}^{2} }}$$

7 $$Z^{\prime} = R_{\text{s}} + R_{\text{ct}} + \sigma_{\text{w}} \omega^{ - 1/2}$$

where *A* is the surface area of the electrode, *c* is the concentration of sodium ions, and *σ*_w_ is the Warburg factor, which can be obtained by calculating the slope of the line *Z*′–*ω*^−1/2^, as shown in Fig. 8b. Table 2 shows that the *D*_Na+_ value of the TiO_2_/N–C NFs (5.8 × 10^−13^ cm^2^ s^−1^) is approximately 2.9 times higher than that of the TiO_2_–C NFs (2.0 × 10^−13^ cm^2^ s^−1^). The increased diffusion coefficient of sodium ions (*D*_Na+_) may be attributed mainly to the incorporation of nitrogen atoms and brings about the better electrochemical performance of the TiO_2_/N–C NF electrode.

**Table 2:** Comparison of EIS parameters of TiO_2_/N–C NFs and TiO_2_/C NFs

Samples	*R*_s_ (Ω)	*R*_ct_ (Ω)	*I*_0_ (mA cm^−2^)	*D*_Na+_ (cm^2^ s^−1^)
N–TiO_2_/C NFs	7.33	85.5	0.30	5.8 × 10^−13^
TiO_2_/C NFs	6.97	210.3	0.12	2.0 × 10^−13^

The Nyquist plots of the TiO_2_/N–C NFs with different amounts of urea are used to explain the causes of these different electrochemical properties (Fig. S7). The simulated results are shown in Table S2. It can be seen that the *R*_s_ values of TiO_2_/N–C NFs with different amounts of urea are similar. However, the *R*_ct_ value of TiO_2_/N–C NFs with 0.1 g of added urea is 85.5 Ω, which is smaller than that of the TiO_2_/N–C NFs with 0.05 g of added urea and much smaller than that of TiO_2_/N–C NFs with 0.2 g of added urea. Hence, it can be deduced that TiO_2_/N–C NFs with 0.1 g of added urea exhibit the smallest electrochemical resistance, indicating the best electron conductivity and electrochemical activity.

## Conclusions

In summary, nitrogen-doped TiO_2_–C composite NFs were fabricated by a facile and green electrospinning method. Inexpensive urea was used as a nitrogen source and pore-forming agent. The as-prepared TiO_2_/N–C NFs exhibited a large specific surface area (213.04 m^2^ g^−1^) and a suitable nitrogen content (5.37 wt%). These characteristics not only contribute to increasing the contact area with the electrolyte and thus shortening the ion/electron diffusion distance, but also essentially enhance the electronic conductivity. As anodes in SIBs, the TiO_2_/N–C NFs exhibit a high reversible capacity (265.8 mAh g^−1^ at 0.05 A g^−1^), an outstanding rate performance (202.4 and 153.7 mAh g^−1^ at 0.2 and 2 A g^−1^, respectively), and an ultra-long cycling durability (118.1 mAh g^−1^ at 5 A g^−1^ after 2000 cycles). This work will open the way to the use of TiO_2_/N–C NFs as one of the most promising anode materials for low-cost SIBs.

## Electronic supplementary material

Below is the link to the electronic supplementary material.

Supplementary material 1 (PDF 723 kb)
