# Supplementary material for: Nitrogen-Doped TiO2–C Composite Nanofibers with High-Capacity and Long-Cycle Life as Anode Materials for Sodium-Ion Batteries
Source: Nanomicro Lett. 2018 Oct 9;10(4):71. doi: 10.1007/s40820-018-0225-1 (PMC6199118; doi:10.1007/s40820-018-0225-1)
Supplement: Supplementary file 1 — Supplementary material 1 (PDF 723 kb) [file 40820_2018_225_MOESM1_ESM.pdf]

Supporting Information for

Nitrogen-Doped TiO_2-C Composite Nanofibers with High-Capacity and Long-Cycle Life as Anode Materials for Sodium-ion Batteries

Su Nie[1], Li Liu[1, 2, *], Junfang Liu[1], Jianjun Xie[1], Yue Zhang[1], Jing Xia[1], Hanxiao Yan[1], Yiting Yuan[1], Xianyou Wang[1]

[1]National Base for International Science & Technology Cooperation, National Local Joint Engineering Laboratory for Key materials of New Energy Storage Battery, Hunan Province Key Laboratory of Electrochemical Energy Storage and Conversion, School of Chemistry, Xiangtan University, Xiangtan 411105, People's Republic of China

[2]Key Laboratory of Advanced Energy Materials Chemistry (Ministry of Education), Nankai University, Tianjin 300071, People's Republic of China

*Corresponding author. E-mail: liulili1203@126.com (L. Liu)

Supplementary Figures

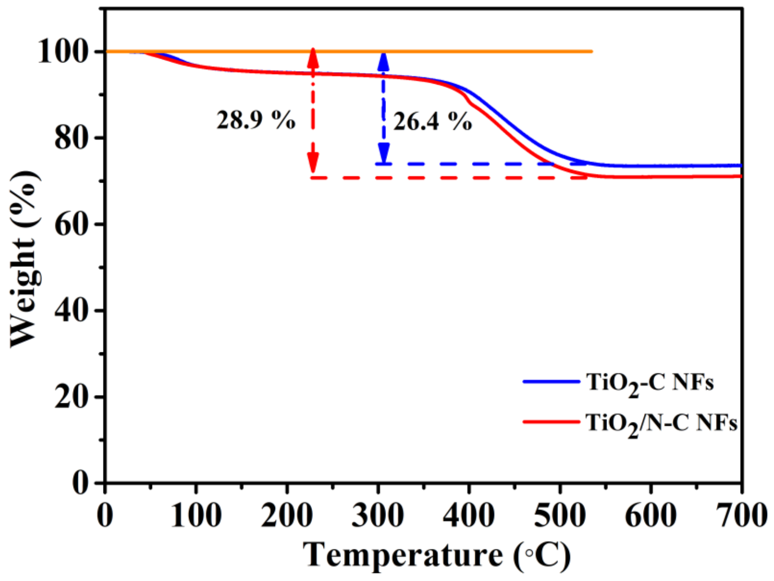

Fig. S1 TGA curves of TiO_2/N-C NFs and TiO_2-C NFs

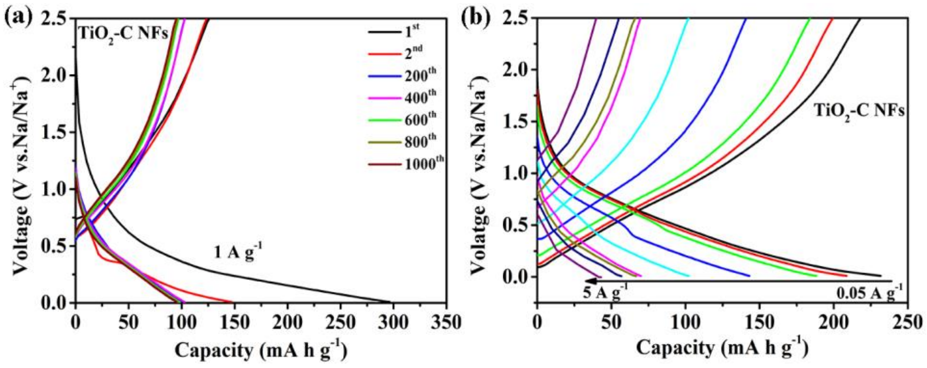

Fig. S2 a Continuous discharge and charge curves of TiO_2-C NFs electrode under a current density of 1 A g^{-1}. **b** Charge-discharge curves of TiO_2-C NFs at 0.05-5 A g^{-1} in the range of 0.01-2.5 V

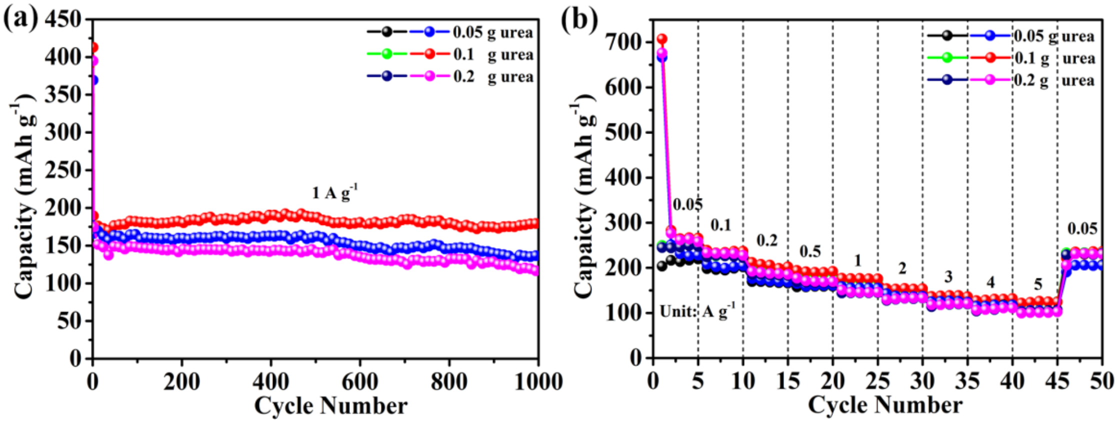

Fig. S3 The electrochemical performances of TiO_2/N-C NFs added with different amounts of urea: **a** Cycle performance at current densities of 1 A g^{-1}. **b** rate capability

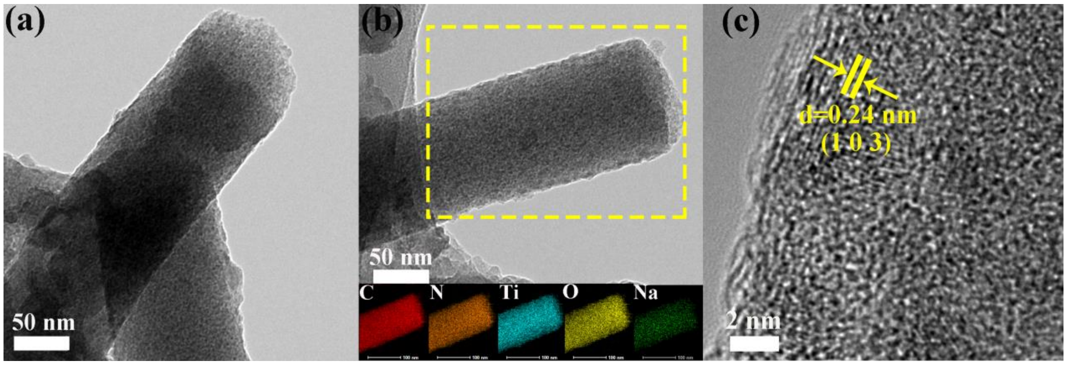

Fig. S4 a, b TEM images and **c** HR-TEM image of the TiO_2/N-C NFs electrode after cycling for 1000 cycles at 1 A g^{-1} in SIBs (the EDS elemental mapping of the area, marked by the yellow rectangle in image **b**)

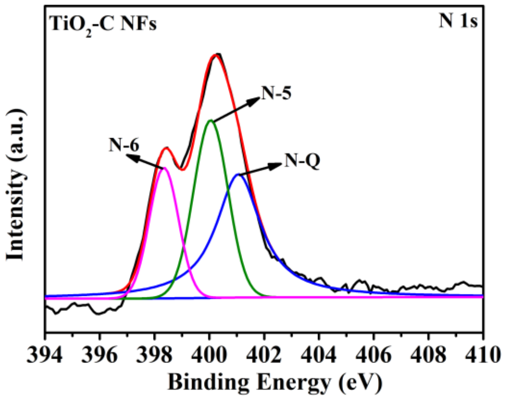

Fig. S5 High-resolution N 1s spectra of TiO_2-C NFs

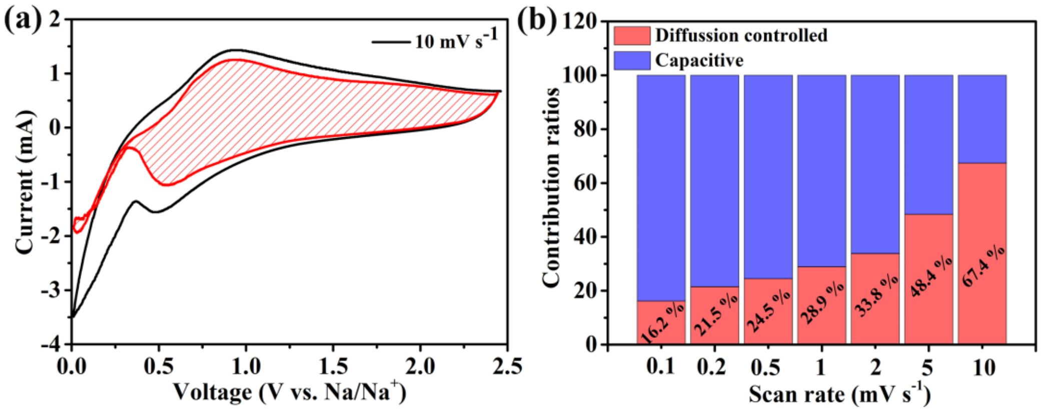

Fig. S6 a Black curve shows the CV curve of TiO_2-C NFs and the red shaded part indicates the capacitive contribution measured at 10 mV s^{-1}. **b** Diagram of capacitive contribution to the total capacity at different scan rate of TiO_2-C NFs

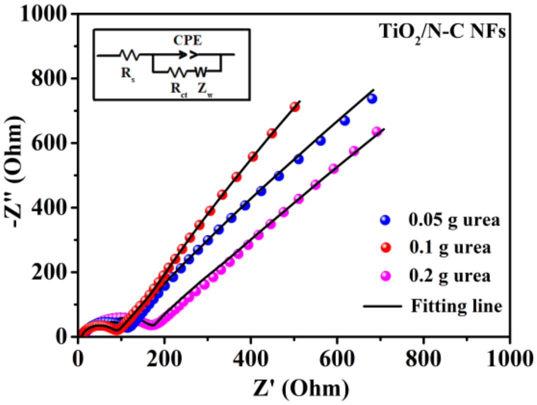

Fig. S7 Nyquist plots and equivalent circuit of the TiO_2/N-C NFs with different amounts of urea in SIBs the first cycle at 0.05 A g^{-1}

Table S1 Comparison of the electrochemical performance of TiO_2/N-C NFs with previously reported TiO_2-based materials as anode in sodium ion batteries

Materials	Rate Performance ($mAh\ g^{-1}$/ $A\ g^{-1}$)	Cycle Performance ($mAh\ g^{-1}$(cycle number)$A\ g^{-1}$)	References
Nitrogen-doped mesoporousTiO_2 Nanofibers	310/0.067<br>108/3.35	110(500th)/3.35	[S1]
Nitrogen-Doped TiO_2 nanospheres	185/0.2<br>156/5	162(1000th)/1	[S2]
Anatase TiO_2@C composites	230/0.033<br>80/6.68	148(500th)/0.5	[S3]
Anatase TiO_2/PVDF	229.8/0.168<br>102.1/6.72	180(500th)/0.335	[S4]
MesoporousTiO_2 nanosheets anchored on graphene	190.8/0.05<br>88.9/1.67	130(2000th)/1.675	[S5]
N-doped carbon coated TiO_2 nanoparticles	204.8/0.168<br>84.9/3.35	122.1(3000th)/3.36	[S6]
Olive-like anatase TiO_2	267/0.336<br>110/6.72	125 (1000th)/3.36	[S7]
Defect-rich $TiO_{2-\delta}$/mooncake-shaped carbon	330/0.05<br>98.1/5	88.5(5000th)/10	[S8]
TiO_2@CNT@C Nanorods	230/0.05<br>115.5/4	153/(1000th)/1	[S9]
TiO_2 particles/carbon	311.5/0.05<br>91.3/6.4	241(500th)/0.4	[S10]
**Nitrogen-doped TiO_2-C composite nanofibers**	**268.5/0.05<br>124.5/5**	**179.2(1000th)/1<br>118.1(2000th)/5**	**this work**

Table S2 Simulated impendence parameters (R_s and R_{ct}) of the TiO_2/N-C NFs with different amounts of urea in SIBs

Samples	0.05 g urea	0.1 g urea	0.2 g urea
R_s (Ω)	7.51	7.33	8.26
R_{ct} (Ω)	104.3	85.5	170.2

Reference

[S1]Y. Wu, X. Liu, Z. Yang, L. Gu, Y. Yu, Nitrogen-doped ordered mesoporous anatase TiO_2 nanofibers as anode materials for high performance sodium-ion batteries. Small **12**, 3522 (2016). https://doi.org/10.1002/smll.201600606

[S2]S. Liu, Z. Cai, J. Zhou, A. Pan, S. Liang, Nitrogen-doped TiO_2 nanospheres for advanced sodium-ion battery and sodium-ion capacitor applications. J. Mater. Chem. A **4**, 18278 (2016). https://doi.org/10.1039/C6TA08472A

[S3]X. Shi, Z. Zhang, K. Du, Y. Lai, J. Fang, J. Li, Anatase TiO_2@C composites with porous structure as an advanced anode material for Na ion batteries. J. Power Sources **330**, 1 (2016). https://doi.org/10.1016/j.jpowsour.2016.08.132

[S4]L. Ling, Y. Bai, Z. Wang, Q. Ni, G. Chen, Z. Zhou, C. Wu, Remarkable effect of sodium alginate aqueous binder on anatase TiO_2 as high-performance anode in sodium ion batteries. ACS Appl. Mater. Interfaces (2018). https://doi.org/10.1021/acsami.7b17659

[S5]R. Zhang, Y. Wang, H. Zhou, J. Lang, J. Xu, Mesoporous TiO_2 nanosheets anchored on graphene for ultra-long life Na-ion batteries. Nanotechnology **29**, 22 (2018). https://doi.org/10.1088/1361-6528/aab562

[S6]J. Wang, G. Liu, K. Fan, D. Zhao, B. Liu, J. Jiang, D. Qian, C. Yang, J. Li, N-doped carbon coated anatase TiO_2 nanoparticles as superior Na-ion battery anodes. J. Colloid Interface Sci. **517**, 134 (2018). https://doi.org/10.1016/j.jcis.2018.02.001

[S7]J. Chen, Y. Zhang, G. Zou, Z. Huang, S. Li, H. Liao, J. Wang, H. Hou, X. Ji, Size-tunable olive-like anatase TiO_2 coated with carbon as superior anode for sodium-ion batteries. Small **12**, 5554 (2016). https://doi.org/10.1002/smll.201601938

[S8]H. He, Q. Zhang, H. Wang, H. Zhang, J. Li, Z. Peng, Y. Tang, M. Shao, Defect-rich $TiO_{2-\delta}$ nanocrystals confined in a mooncake-shaped porous carbon matrix as an advanced Na ion battery anode. J. Power Sources **354**, 179 (2017). https://doi.org/10.1016/j.jpowsour.2017.04.035

[S9]Y.E. Zhu, L. Yang, J. Sheng, Y. Chen, H. Gu, J. Wei, Z. Zhou, Fast sodium storage in TiO_2@CNT@C nanorods for high-performance Na-ion capacitors Adv. Energy Mater. **7**, 1 (2017). https://doi.org/10.1002/aenm.201701222

[S10] H. Tao, M. Zhou, K. Wang, S. Cheng, K. Jiang, Glycol derived carbon- TiO_2 as low cost and high performance anode material for sodium-ion batteries Sci. Rep. **7**, 43895 (2017). https://doi.org/10.1038/srep43895
